# Contribution of Auto-Visual AFP Detection and Reporting (AVADAR) on polio surveillance in South Sudan

**DOI:** 10.11604/pamj.supp.2022.42.1.33788

**Published:** 2022-06-17

**Authors:** Ayesheshem Ademe Tegegne, Sylvester Maleghemi, Evans Mawa Oliver Bakata, Atem Nathan Anyuon, George Awzenio Legge, Anthony Laku Kibrak, Johnson Muluh Ticha, Daudi Peter Manyanga, Isah Mohammed Bello, Kibebu Kinfu Berta, Fabian Ndenzako, Mkanda Pascal, Olushayo Oluseun Olu

**Affiliations:** 1 WHO, South Sudan Country Office, Ministerial Complex, Juba, South Sudan,; 2 Ministry of Health, Ministerial Complex, Juba, South Sudan,; 3 WHO, Regional Office, Brazzaville, Congo,; 4 Inter-Country Support Team, Harare, Zimbabwe

**Keywords:** Acute flaccid paralysis surveillance, AVADAR, South Sudan, polio, short message service, community informant

## Abstract

**Introduction:**

the last wild polio virus in South Sudan was documented in 2009. Nonetheless, it was one of the last four countries in the WHO African region to be accepted as a polio-free country in June 2020. In line with this, to accelerate the polio-free documentation process, the country has piloted Auto Visual AFP Detection and Reporting (AVADAR) in three counties. This study examined the contribution of the AVADAR surveillance system to the traditional Acute Flaccid Paralysis (AFP) surveillance system to document lessons learnt and best practices.

**Methods:**

we performed a retrospective descriptive quantitative study design to analyze secondary AVADAR surveillance data collected from June 2018 to December 2019 and stored at the WHO AVADAR server.

**Results:**

the AVADAR community surveillance system has improved the two main AFP surveillance indicators in the piloted counties and made up 86% of the total number of true AFP cases detected in these counties. The completeness and timeliness of weekly zero reporting were 97% and 94%, respectively and maintained above the standard throughout the study, while the two main surveillance indicators in the project area were improved progressively except for the Gogrial West County. In contrast, main surveillance indicators declined in some of the none-AVADAR implementing counties.

**Conclusion:**

the AVADAR surveillance system can overcome the logistical and remoteness barriers that can hinder the early detection and reporting of cases due to insecurity, topographical, and communication barrier in rural and hard-to-reach areas to accomplish and sustain the two main surveillance indicators, along with the completeness and timeliness of weekly zero reporting. We recommend extending this application-based surveillance system to other areas with limited resources and similar challenges by incorporating other diseases of public health concern.

## Introduction

Surveillance is one of the four strategies for the global polio eradication initiative recommended by the World Health Organization (WHO). It has been proven effective in many countries that have successfully eradicated polio [1,2]. Surveillance for polio eradication heavily relies on immediate detection and notification of acute flaccid paralysis cases with a follow-up investigation and testing stool specimens in WHO accredited laboratories [3]. This determines where, when, and how Wild Polio Virus (WPV) is circulating to take appropriate response activities. It also reliably shows where transmission had been interrupted [4]. Nonetheless, in practice maintaining a high-quality AFP surveillance system is challenging in hard-to-reach and insecure areas as it is difficult routinely to access and conduct active case search, which leaves a blind spot in the area [5,6]. The country´s security crisis has contributed to a significant ongoing humanitarian crisis, with an estimated 1.67 million internally displaced persons and 2.4 million refugees leaving for safety in other countries. At the same time, 7.5 million need urgent humanitarian assistance as of October 2019 [7,8]. Years of protracted conflict, poverty, and socio-economic marginalization in South Sudan have left a significant negative impact on the population´s health, leaving the health care system in much worse conditions, which unable to address the growing and diverse needs of the people. This situation is exacerbated by the harsh climate conditions resulting in a prolonged rainy season with floods. As a result, many counties are cut off and inaccessible for over six months, adding challenges to already fragmented health services [9-11].

Like many other countries, South Sudan´s surveillance system is placed around the health facility, provided that individuals with complaints will come to the health facility, and the attending health worker will detect any suspected acute flaccid paralysis (AFP) case. Nevertheless, most of the population in South Sudan lives in rural areas with limited access to health services on top of frequent population movement, displacement and interclan conflicts [12]. Auto-Visual AFP Detection and Reporting (AVADAR) is one of the innovative mobile-based technological interventions deployed in areas with unique challenges. The use of Android smartphones has been documented in many countries in Nigeria, and Lake Chad in public health programs to improve disease surveillance and immunization [5,13]. Auto visual AFP detection and reporting was first piloted in Nigeria and found to be effective in detecting and responding to cases in areas with limited access. Following this, AVADAR has been introduced in other African countries to accelerate the regional certification process [14]. In line with this, in June 2018, South Sudan introduced- AVADAR in three counties in Gogrial West, Juba, and Terekeka, spanning two states of Central Equatoria and Warrap. This study aims to examine the contribution of the AVADAR community surveillance system to the traditional AFP surveillance system, and compare with other non-AVADAR implementing counties and document lessons learned and best practices.

## Methods

**Study design:** we conducted a retrospective descriptive quantitative study design using the secondary AVADAR surveillance data collected from June 2018 to December 2019 and stored at the WHO AVADAR server.

**Setting:** the AVADAR surveillance program was established in June 2018 in three counties of Central Equatoria and Warrap states. The population of the States of Central Equatoria and Warrap is estimated to be 1,784,053 and 1,560,963, respectively (projected from the 2008 census). Central Equatorial and Warrap states consist of six and seven counties, respectively. Overall, there are 25 Payams in the piloted counties. The total population of the three pilot counties is estimated to be 1,237,894, while 582,327 are under 15 years of age. Cellular network service in these counties is provided by two telecommunication operators (i.e., MTN and Zain), while remote villages within the county may be challenging to access the network. Terekeka and Juba can be accessed by road from the national level, while a flight only accesses Warrap.

**Study population:** the study population includes all documented AFP cases among children under 15 years of age that have been notified by the community informants and confirmed by WHO surveillance officers as TRUE AFP cases. Any case that has not been confirmed for whatever cause was excluded.

**Training of participants:** at the beginning of the program, on average, 10 Community Informants (CI) were selected from 25 Payams and trained for an initial three days conducted in small groups about the principles of AVADAR application. Demonstrations, group work, and role-play sessions were used so that the CIs effectively managed the AVADAR application. A 1-day additional training was also conducted for coordinators and technical officers on data management and operational issues. At the end of the training, each participant was provided with an Android phone with the AVADAR application with SIM and a portable solar charger. Technical officers managed all phone trouble shootings from eHealth assigned at each Payam throughout the study period. Senior facilitators conducted the training from WHO, Novel T, and eHealth Africa.

**Figure 1:**
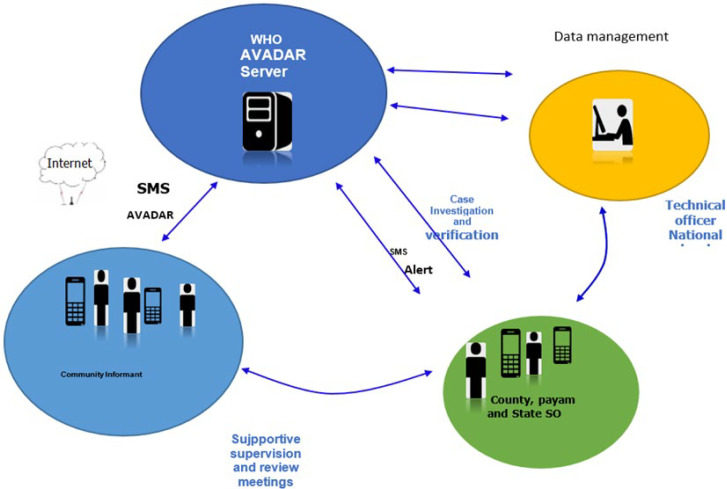
AVADAR community-based surveillance information and flow system 2018-2019, South Sudan

**The AVADAR surveillance process:** AVADAR is a mobile-based technology used by community informants to detect and notify suspected cases of AFP directly from the community. The application performs four operations: i) case reporting via Short Message Service (SMS) by the CI, as well as weekly zero reports; ii) the server generates an automatic SMS to the Payam surveillance officer; iii) an automatically generated email containing the investigation’s findings; iv) save data, including the dashboard, to the server. Additionally, the application has embedded video that becomes live every Monday at precisely 11:00 AM. A video with a detailed description of an AFP case runs and asks whether there is an AFP (“Yes”) and has not been reported or to confirm that the informant has not seen any AFP cases (“No”) throughout the week (weekly zero reports). If the CI sends a ”Yes” SMS with brief data, then the server will automatically trigger an alert to the cell phone of the corresponding Payam Surveillance officer with a short description of the case. The CIs use the running video also to conduct sensitization of the community. Upon receiving notification, the Payam surveillance officer investigates and sends the result of the investigation to the server by filling out the necessary information using the Open Data Kit (ODK). This includes the Report Submission ID (RSID) that allows linking the data collected by CI. Once the result is received, the server-generated SMS will automatically share the result of the investigation with all parties involved in the program. If the case is a true AFP case, the county field supervisor collects and transports specimens per the national guidelines. At the same time, the Payam officer and the national coordinator conduct supportive supervision and monthly review meetings in collaboration with county coordinators (Figure 1). For communication, a contract was made with each of the two cellular network providers (MTN and Zain) as appropriate to allow free voice communication and upload data. The Android phones are loaded with top-ups, data bundles, and a closed user caller group that enable cheap calls between the personnel involved.

**Data collection methods and analysis:** we used secondary AVADAR AFP surveillance data uploaded daily on the WHO AVADAR server between June 2018 and December 2019. All AFP cases detected and reported within the study period were included. We excluded all cases that did not meet the case definitions and cases >60 days to comply with the national AFP guideline. Based on the national database for AFP, we calculated the average performance of the main surveillance indicators of none of the three counties covered by AVADAR. We then compared the result with the AVADAR implementing counties from 2014-2019. These non-AVADAR implementing counties have almost comparable characteristics to those implementing AVADAR as they are neighboring counties within the states implementing AVADAR surveillance. For further analysis, we also compared the surveillance performance of the AVADAR implementing counties before and during the implementation period. The completeness and timeliness of zero weekly reports were also compared with the set standard. The national coordinator for AVADAR regularly cleans the data, while the system automatically disables some wrong entries. Frequencies, tables, and graphs were produced using MS Excel, and the results of the analysis were compared with the surveillance performance standards set by the WHO.

### Definitions of terms

**Active informants:** informants that reported a suspected AFP case or sent a “zero report” when no case was seen within the reporting week.

**AVADAR:** Auto-Visual-Acute Flaccid-Paralysis Detection and Reporting.

**Community Informants (CIs):** a resident of the community volunteered to be an informant for AFP.

**Timeliness of weekly zero reporting:** number of zero reports submitted on time.

**Completeness of weekly zero reporting:** total number of reporting units submitted weekly zero reports.


$$Non-polio\text{ AFP rate=}\frac{Number\text{ of n}on-polio\text{ AFP cases < 15 years old }}{\text{total number of children < 15 years old}}\times 100000$$

$$Stool\text{ Adequacy=}\frac{Total\text{ number of cases with 2 stool specimen collected within 14 days of onset of paralysis and good condition}}{Total\text{ AFP cases reported}}\times 100000$$

Ethical approval and consent: administrative clearance for publication of this manuscript was provided by the Ministry of Health of South Sudan and WHO under the executive clearance (ePub-IP-00331583-EC). Moreover, the Research Ethics Review Board of the Ministry of Health provided clearance for the publication of the manuscript (MoH/RERB/D.03/2022). We used the secondary data collected and stored at the AVADAR server. Individual verbal consent was received during stool specimen collection and filling of the required information in the case-based form.

## Results

**Figure 2:**
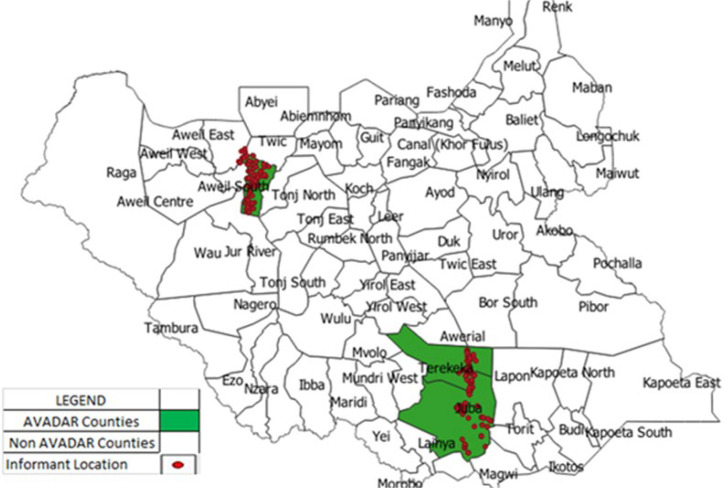
AVADAR implementation area 2018-2019, South Sudan

Over the period of analysis, the information generated by the community informants followed and responded by the Payam Officer with close supervision and monitoring by the county Field supervisor. On the other hand, the national coordinator has managed the data and gave regular feedback to the county officers and to all concerned (Figure 1). After 21 months of the pilot study, the number of community informants that were active and sending weekly zero reports remained at 234 (93%) of the initially trained community informants. The majority of 179 (77%) of the community informants are male, 221 (94%) are literate, and 229 (98%) had lived in the Payam for > 5 years (Table 1). At the begging of the project, overall, 251 community informants were engaged and distributed along the 25 study Payams in 3 counties (Figure 2). Of the total of 20,210 weekly SMS zero reports expected over the 21 months, 19,011 weekly zero reports were received, giving completeness of weekly zero reporting of 97%, while 19,019 (94%) of the weekly zero reports were received timely. All counties achieved the set target of 80% for timely reporting and 90% for completeness of weekly zero reporting, increasing in 2019 compared to 2018 (Table 2). A total of 578 alerts for suspected cases of AFP were notified by community informants, of which 577 (99.7%) were investigated within 48 hours of notification by the Payam officers (ranged from 96% to 97%) in all the project counties. Of those suspected cases notified by community informants and investigated by officers in the three pilot counties, 56 (11%) of the cases were true AFP cases, contributing to 86% of the total AFP cases reported in the counties implementing the AVADAR surveillance system. In Terekeka and Gogrial West, the contribution of AVADAR was 97% and 100% of the cases reported, respectively, while the lowest (60%) contribution was documented in Juba County. In AVADAR implementing counties, the average number of days between the date of onset and date of detection of cases was 5 days compared to 8 days in the traditional surveillance system throughout the country (Table 2).

**Table 1:** number of community informants and project catchment population, South Sudan 2018-2019

Name of implementing County	No of implementing Payams	Community informants at the start of the project	Active informants at December 2019	% Community informants active	Sex		Education		% CIs living in the Payam	
					Male	Female	Literate	Literate	>5 years	< 5 years
Juba	10	97	84	87.6	62	22	85	0	82	3
Terekeka	6	59	54	91.5	48	6	41	13	52	2
Gogrial west	9	95	95	98.9	69	26	95	0	95	0
Total	25	251	233	92	179	54	221	13	229	5

**Table 2:** characteristics of AVADAR implementing counties, South Sudan, June 2018-Dec 2019

Description	Juba	Terekeka	Gogrial West	Total
Weekly report expected	7737	4719	7754	20210
weekly report received	7390	4569	7552	19511
% completeness	96	97	97	97
Weekly report expected	7737	4719	7754	20210
Report received timely	7255	4415	7349	19019
% timeliness of reporting	94	94	95	94
Suspected alerts total	322	127	129	578
Alerts investigated	321	127	129	577
% of AFP investigated with 48 hrs	100	100	100	100
AFP cases reviewed by external team				
Number of AFP cases reviewed	9	8	26	43
Number of true AFP cases	9	5	20	34
% true AFP cases	100	62	77	79
AVADAR reported cases				
Total AVADAR AFP cases	12	10	34	56
No. discarded as false AFP cases	309	117	95	521
% true AFP cases	4	9	36	11
Total non AVADAR AFP cases	8	1	0	9
Total AFP cases in the county	20	11	34	65
% AFP cases reported through AVADAR	60	91	100	86
Average days between onset and detection	4	5	6	5

Unlike other counties in the traditional surveillance, AVADAR implementing counties registered high timeliness of reporting, including investigation of cases with 48 hours

**Figure 3:**
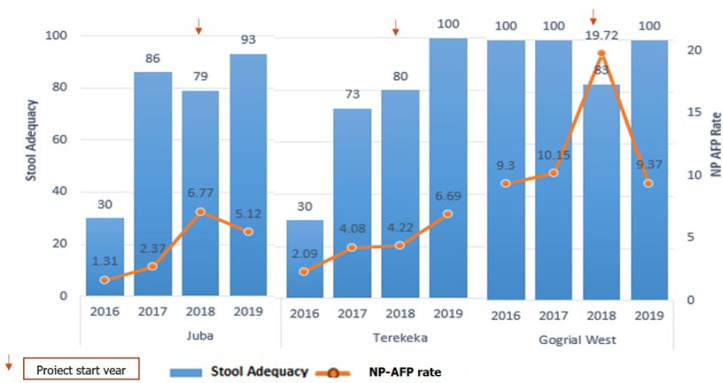
main surveillance indicators in AVADAR implementing counties, June 2018- Dec 2019

**Figure 4:**
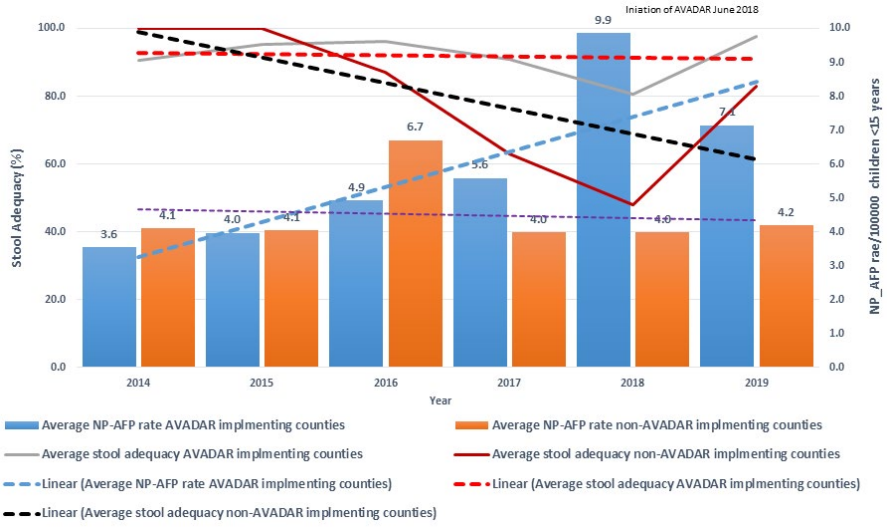
comparison of average two main surveillance indicators in AVADAR and non-AVADAR implementing counties 2014-2019

Before implementing AVADAR, almost all counties in the project area did not meet the two main surveillance indicators, except for Gogrial West. After the introduction of AVADAR, all counties showed a progressive improvement in the two main AFP surveillance performance indicators, most notably in Juba and Terekeka. In contrast, Gogrial West showed an increase immediately at the start of the pilot but declined and maintained the same level as before the implementation period (Figure 3). AVADAR implementing counties showed a positive linear trend of the NP-AFP rate during the pilot. Non-AVADAR implementing counties remained flat throughout the pilot. On the contrary, the stool adequacy in AVADAR implementing counties showed a flatter trend above the certification standard (80%). In comparison, non-AVADAR implementing counties showed a progressive decline in stool adequacy below the standard of stool adequacy of 80% (Figure 4). Over the study period, 567 cases of diseases of public health importance were reported through the system by the community informants. The most important reported conditions were measles, meningitis, watery diarrhea, malnutrition, and malaria (Table 3).

**Table 3:** other vaccine preventable disease reported through AVADAR community informants June 2018-Dec 2019

County	Number of suspected measles cases	Number of suspected watery diarrhea	Number of suspected protein/energy malnutrition	Number of suspected malaria cases	Number suspected Guinea worm cases
Juba	3	28	57	128	0
Terekeka	2	10	15	43	0
Gogrial West	28	45	67	139	2
Total	33	83	139	310	2

## Discussion

The results of our study demonstrated that the two main surveillance indicators in the pilot counties are improved, and are in line with the results found in Nigeria, according to which AVADAR increased main surveillance indicators as well as detection and notification of cases [13]. This finding is also consistent with other countries´ national surveillance performance indicators [15-17]. Nonetheless, within the project counties, the results are variable. In Juba and Terekeka counties, there had been a steady increase in the NP-AFP rate, whereas, in Gogrial West, was not persistent. In the begging, it started to pick up but later it dropped to a pre-implementation level. On the other hand, stool adequacy improved in all counties. This finding supports the statement that the traditional surveillance of case detection in Gogrial West functioned relatively well even before introducing the AVADAR system. Following the introduction, the surveillance system has become more sensitive, and probably more AFP cases may have been reported beyond 2 weeks of onset due to over-sensitization.

We found a higher rate of completeness and timeliness of weekly zero reporting in the piloted countries, unlike the results of the national, traditional AFP surveillance report and the Integrated Disease Surveillance and Response weekly report [18]. However, our finding compares favorably with those of Faisal et al. in Nigeria, where surveillance reporting increased following the AVADAR introduction, and the WHO recommended standard for zero reporting [13,19]. The high completeness and timeliness of zero reporting in our study may have been facilitated by adding a video application embedded in the AVADAR system. The video application automatically runs every week to remind the community informants to send weekly zero reports. Furthermore, the high report may be simplified with close supervision, monitoring, and coaching of informants on top of regular monthly review meetings. On the other hand, the highest percentage of AFP cases investigated within 48 hours ties well with the WHO-recommended surveillance standard for the investigation of suspected AFP cases [19]. The high performance of our findings may have also been fueled by the presence of a fast SMS notification system by CIs and an automated alert system from the server to the Payam surveillance officers. It may also be simplified by the presence of a Payam surveillance officer in each Payam who investigates immediately once he receives an alert from the server, in which case communication, logistic, and access challenges are minimized, unlike the traditional system is suffering.

Different diseases of public health importance, including diseases under global eradication, were notified through the community informants on top of the beneficial effect of AVADAR on the improvement of the two main surveillance indicators by widening the geographic areas of case detection, resulting in an increase in case reporting and the scope of diseases reported [20]. Although many positive results were documented in implementing the AVADAR surveillance system, there were challenges in implementing the pilot. This includes poor network coverage in some study areas, which necessitated CIs walking long distances to areas where the network is good. Lack of power to regularly charge the phones due to damage to solar charges and lack of other phone accessories. However, the staff made frequent follow-ups and immediate remedies to resolve these issues.

One of the limitations of this study is that a baseline study was not done before the project’s initiation to compare the results pre-and post-implementation of the pilot. However, we used the pre-pilot performance indicators to compare with current achievement both with implementing and none implementing counties, which was well documented in the AFP surveillance system. Despite these limitations, we noted that the use of AVADAR, coupled with the traditional routine surveillance system, has contributed to the successes in meeting main surveillance indicators.

## Conclusion

Our study demonstrated that AVADAR could overcome the logistical and distance barriers that can impede the early detection and reporting of cases in rural and hard-to-reach areas to attain the two main surveillance indicators, along with the completeness and timeliness of weekly zero reporting. We recommend expanding the AVADAR surveillance system into other poor-performing counties with similar challenges. However, before this, an in-depth baseline assessment should be conducted, focusing on surveillance indicators, network availability, and access to power to charge phones. We also suggest integrating other diseases of public health importance into the AVADAR surveillance system by incorporating locally appropriate tools that community informants can use during active surveillance, sensitization, and reporting. This platform could also be scaled up to cover the most critical child survival initiatives like immunization (defaulter tracking) and Maternal Death Surveillance (MDS).

### What is known about this topic

- AVADAR is known to increase the sensitivity of AFP surveillance in hard-to-reach and conflict-affected areas by using trained community informants;
- Acute Flaccid Paralysis (AFP) is the primary strategy for polio eradication, AVADAR supplements the traditional surveillance system by extending the surveillance network to the community level using SMS mobile-based technology and trained community informants;
- Moreover, information is sent automatically to the designated officer when the community informant finds a suspected case; this technology helps to improve the completeness, timeliness, and availability of AFP reporting.

### What this study adds

- The study has systematically evaluated the use of AVADAR in South Sudan for the first time in hard-to-reach counties with poor surveillance performance and compared it with non-AVADAR implementing counties in terms of main surveillance indicators;
- The analysis of this study also finds out that AVADAR can also be used to monitor other vaccine-preventable and child survival initiatives;
- There is a significant increase in the main surveillance indicators in the piloting counties post AVADAR implementation compared to non-AVADAR counties.program reporting performance, thus allowing detection of major outbreaks, and averting morbidity and mortality;
- Program funding from domestic resources is critical for sustainability in the long-term.
